# Cognition in Treatment Resistant versus Non- Treatment Resistant Schizophrenia

**DOI:** 10.1192/j.eurpsy.2025.1878

**Published:** 2025-08-26

**Authors:** S. Shah, K. Patel, A. Bhandari, A. Porwal, N. Lalwani

**Affiliations:** 1 B.J. Medical College, Ahmedabad, India

## Abstract

**Introduction:**

Despite antipsychotic treatment, around one-third of individuals of schizophrenia remain unresponsive.

**Objectives:**

Comparing cognitive impairments in TRS versus non-TRS patients.

**Methods:**

50 adult patients with schizophrenia(DSM-5) were recruited in this cross-sectional cohort study, categorised into TRS (14) (treatment-resistant schizophrenia) and NTRS (36) (non-treatment-resistant schizophrenia) by modified Kane criteria. Positive and Negative Syndrome Scale (PANSS) and Montreal Cognitive Assessment Scale (MoCA) were used.

**Results:**

**Table:** 

Table 1	TRS	NTRS	Overall
Male(%)	42.8	63.8	58
Female(%)	57.1	36.1	42
AGE (years)	41.7 ± 13.1	41.4±12.3	41.5±12.4
DURATION OF ILLNESS(years)	17.2±10.8	6.3±6.8	9.4±9.4

**Table:** 

Table 2	TRS	NTRS	Independent sample students’ T test pvalue
MoCA	19.2±6	27.8±5.4	<0.01
TOTAL PANSS	106.3±22.4	57.4±21.	<0.01
POSITIVE PANSS	22.1±7.3	13.8±6.4	<0.01
NEGATIVE PANSS	26.8±5.7	14.1±5.1	<0.01
Generalised PANSS	57.3±13.2	29.4±11.4	<0.01
COMPOSITE SCORE	-4.71±7.6	-0.2±5.6	0.02

MoCA Scores (Table 2) are significantly lower in the TRS group, implying NTRS has a moderate cognitive decline, and TRS has severe cognitive decline. Total PANSS, Positive, Negative,and Generalised PANSS are significantly lower in the TRS group indicating severe symptoms than NTRS.Classifying based onTotal PANSS Score, the TRS has severely ill, while the NTRS has borderline mentally ill patients. The composite score indicates that the TRS group tends to have more negative symptoms. Computing the data’s variances and independent sample Welch’s t-test (Image 3) for data with unequal variances and independent sample student’s test (Images 1, 2) for data with equal variances were performed. Age does not significantly affect TRS(Table 1). Duration of illness is significantly higher in the TRS group. MoCA domains (i.e., executive function, visuospatial, orientation, and attention) except memory and language have significantly lower scores in the TRS group. Positive symptoms (conceptual disorganisation, excitement,grandiosity, suspicion, and hostility) except delusion and hallucinatory behaviour were significantly higher in the TRS group. Negative symptoms (emotional withdrawal, poor rapport, passive/apathetic social withdrawal, difficulty in abstract thinking, lack of spontaneity and flow of conversation, stereotype thinking) except blunted effect were significantly higher in the TRS group. Generalised symptoms (somatic concern, anxiety, guilt feelings, mannerism and posture, depression, motor retardation, uncooperativeness, unusual thought content, social disorientation, poor attention, lack of judgement, disturbance of volition, poor impulse control, preoccupation, and active social avoidance) except tension were significantly higher in the TRS group.

**Image:**

**Figure:**
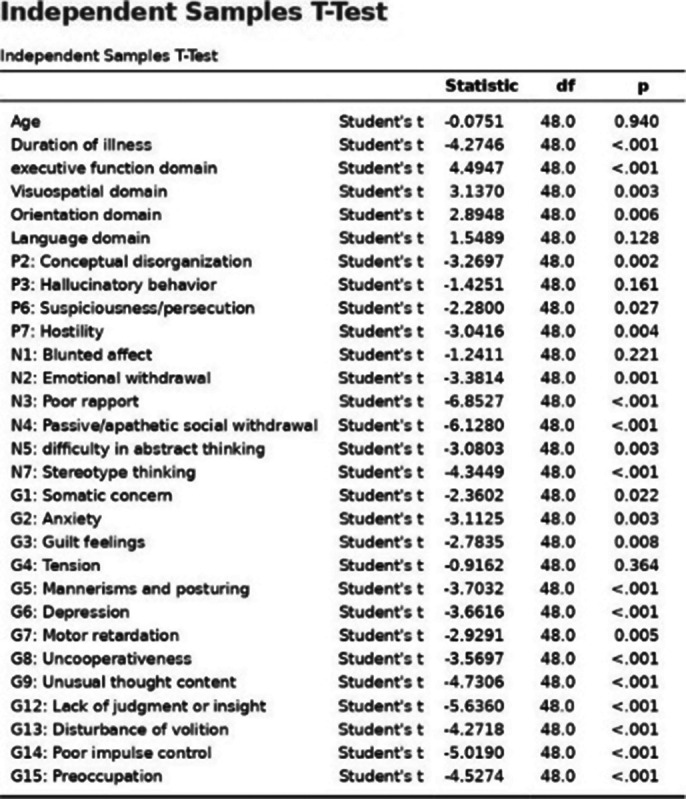

**Image 2:**

**Figure:**
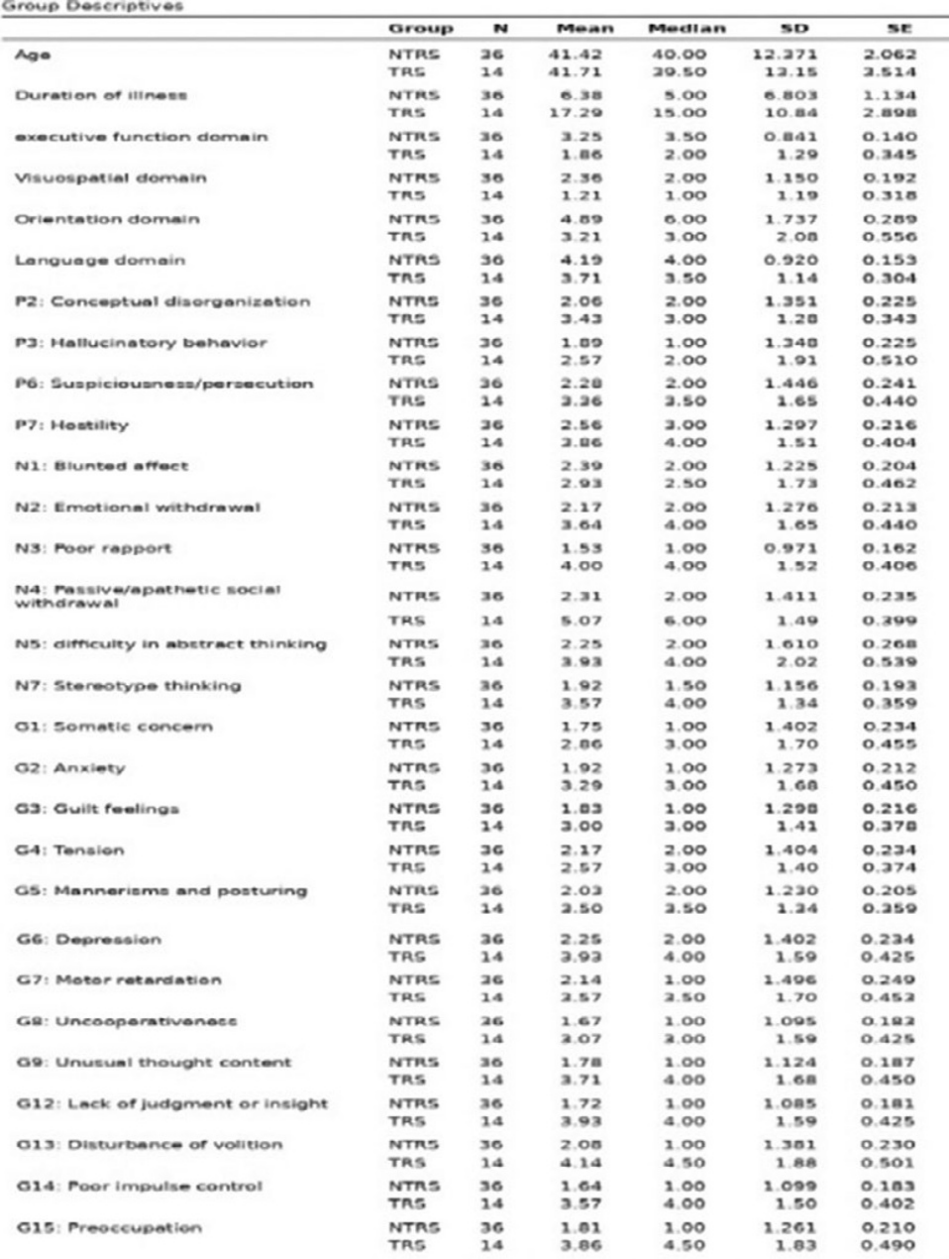

**Image 3:**

**Figure:**
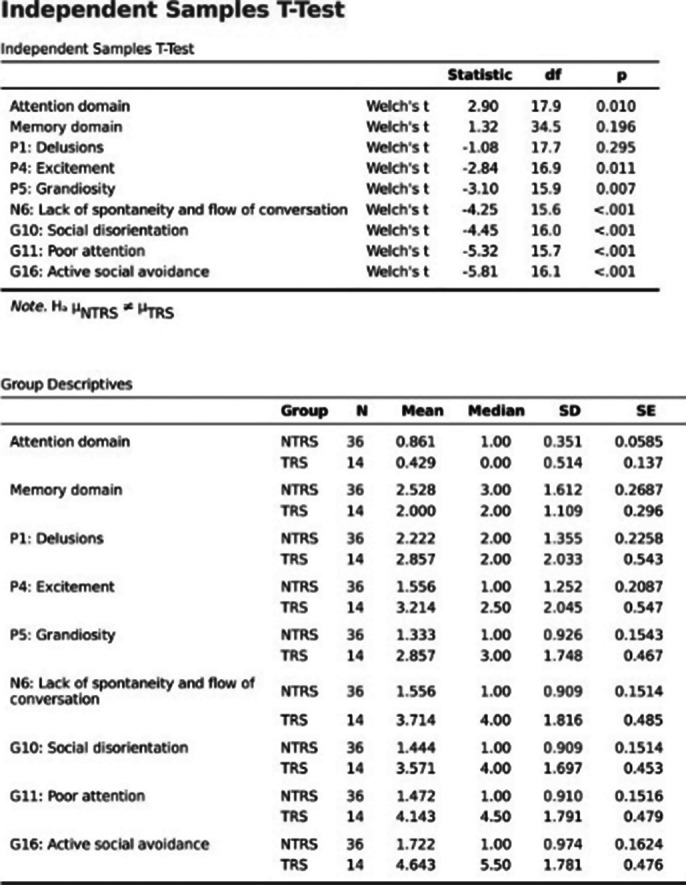

**Conclusions:**

TRS patients show severe cognitive impairment, however it does not impact language and memory. TRS shows more symptom severity except delusion, hallucinatory behaviour, blunted effect, and tension as compared to NTRS.

**Disclosure of Interest:**

None Declared

